# Functional THz emitters based on Pancharatnam-Berry phase nonlinear metasurfaces

**DOI:** 10.1038/s41467-020-20283-0

**Published:** 2021-01-04

**Authors:** Cormac McDonnell, Junhong Deng, Symeon Sideris, Tal Ellenbogen, Guixin Li

**Affiliations:** 1 Department of Physical Electronics, School of Electrical Engineering, Tel-Aviv University, 6997801 Tel Aviv, Israel; 2 Center for Light-Matter Interaction, Tel-Aviv University, 6779801 Tel-Aviv, Israel; 3 Department of Materials Science and Engineering, Southern University of Science and Technology, 518055 Shenzhen, China; 4 Shenzhen Institute for Quantum Science and Engineering, Southern University of Science and Technology, 518055 Shenzhen, China

**Keywords:** Nanophotonics and plasmonics, Metamaterials, Nonlinear optics

## Abstract

Recent advances in the science and technology of THz waves show promise for a wide variety of important applications in material inspection, imaging, and biomedical science amongst others. However, this promise is impeded by the lack of sufficiently functional THz emitters. Here, we introduce broadband THz emitters based on Pancharatnam-Berry phase nonlinear metasurfaces, which exhibit unique optical functionalities. Using these new emitters, we experimentally demonstrate tunable linear polarization of broadband single cycle THz pulses, the splitting of spin states and THz frequencies in the spatial domain, and the generation of few-cycle pulses with temporal polarization dispersion. Finally, we apply the ability of spin control of THz waves to demonstrate circular dichroism spectroscopy of amino acids. Altogether, we achieve nanoscale and all-optical control over the phase and polarization states of the emitted THz waves.

## Abstract (web summary)

The enormous application potential of THz waves demands for precise control of THz pulse generation. Here, the authors present a nonlinear metasurface that enables tunable linear polarized few cycle THz pulses.

## Introduction

Increasing numbers of important industrial and scientific applications aim to employ terahertz (THz) electromagnetic waves^1–4^. Their relatively low energies allow probing of vibrionic and rotational transitions in molecules, which makes them useful to remotely identify a wide variety of material compositions and structures^5^. Furthermore, their penetration into optically opaque materials can be applied to remote inspection of concealed areas for security^6^ or inspecting circuits in electronic devices^7^, and even studying hidden layers in paintings^8^. Moreover, they can also be used as non-invasive and non-ionizing radiation sources for early detection of numerous medical conditions, such as skin cancer^9^ and cavities in teeth^10^. In addition, the use of circularly polarized light sources across various regions of the electromagnetic spectrum, as well as the THz frequency band, has attracted interest for applications of circular dichrosim spectroscopy^11^. This field of spectroscopy has found widespread interest owing to the abundant amount of chiral molecules and materials, which are readily found in nature, from proteins to amino acids. This application is especially relevant for the THz frequency band, which has many strong absorption peaks for organic molecules. However, generating functional broadband circularly polarized light in the THz regime still poses many challenges^12^. Therefore, over the past three decades, a great amount of research has been devoted to the science and technology of THz waves. These efforts have advanced the ability to generate, control, and detect THz waves through numerous methods^13–17^. Yet, state-of-the-art THz emitters still do not have the capabilities and functionalities of their counterparts in the radio frequency, microwave, and optical regimes. This limitation has strongly frustrated the widespread deployment of emerging THz technologies and their applications.

In the field of photonics, the past decade has witnessed a revolution spurred by the extensive and rapid development of optical metamaterials and metasurfaces^18–20^. These innovations consist of engineered subwavelength building blocks, usually composed of metal or high-index dielectric meta-atoms, ordered in various controlled geometries. Their optical properties can be tailored by understanding both the interaction between light and an individual building block and the collective coupling dynamics over the entire metasurface. Unprecedented control of the amplitude, polarization, and phase response of light can be achieved at subwavelength lateral resolution using deep-subwavelength thin films. These control capabilities of metasurfaces have been viably demonstrated for a wide range of applications including, for example, ultrathin achromatic optics^21^, optical holography^22^, polarization control^23,24^, quantum entanglement^25^, and the construction of reconfigurable optical elements^26^. Furthermore, in the nonlinear optical regime, applications such as frequency conversion, nonlinear beam shaping, and nonlinear optical holography have been successfully demonstrated with the metasurface platforms^27,28^. Recently, THz generation using metasurfaces was also demonstrated, opening the door to the precise control of THz waves^29–32^. However, these preliminary demonstrations do not fully exploit the potential of metamaterials for generating and actively controlling THz waves, and breakthroughs in the development of the next generation of THz emitters await.

Here we demonstrate a new kind of metasurface-based THz emitter, in which both the polarization and phase of THz waves can be finely controlled by using the concept of geometric Pancharatnam-Berry (P-B) phase, in the nonlinear optical regime. The building block of the proposed emitter is a nanoscale gold plasmonic meta-atom, which has three-fold (C3) rotational symmetry. We show that under illumination with fundamental waves (FWs) having both left and right circular polarizations (LCP and RCP), the C3 meta-atom emits THz waves with spin-dependent geometric phases. Unlike the nonlinear geometric P-B phase demonstrated in harmonic generation processes, in which the polarization states of fundamental waves can be either LCP or RCP, here the fundamental waves must contain both circular polarization states, which allows excitation with linearly polarized light. Using spatially variant meta-atoms on the plasmonic metasurface, the phase and polarization of the THz waves can be controlled at a deep-subwavelength scale. This enables the realization of THz emitters with exciting new functionalities, as we demonstrate later on.

## Results

### Selection rules for THz generation using C3 meta-atoms

The geometric Berry phase has played a major role in explaining many important phenomena in condensed matter physics^33^. In the field of optics, it is known as the Pancharatnam-Berry (P-B) phase^34,35^, and enables the introduction of topologically varying phase differences to tailor a desired output field. This has been a key concept in the development of optical metasurfaces for manipulation of light in both the linear and nonlinear regimes. The concept of geometric phase in nonlinear harmonic generation processes on plasmonic metasurfaces was first proposed and studied recently^36^. It was found that the phase of the generated harmonics could be determined and controlled by the spin states of the fundamental wave, the order of harmonic generations, and the rotational symmetry of the meta-atoms, and these findings collectively introduce new means of controlling harmonic generation by nonlinear metasurfaces^37^. In the case of broadband THz generation from metasurfaces, second-order optical rectification was suggested as a potential underlying mechanism^31^. In this process, frequency components of the exciting pulse interact through the structural nonlinearity of the meta-atoms. This interaction generates THz waves at $\omega _{\text{THz}} = \omega _1 - \omega _2$, where $\omega _1$ and $\omega _2$ are two discrete frequencies within the bandwidth of the pump pulse. The nonlinear polarization term that is driven by the two frequencies is given by $P\left( {\omega _{{\mathrm{THz}}}} \right) = \varepsilon _0\chi ^{\left( 2 \right)}E\left( {\omega _1} \right)E \ast (\omega _2)$^38^, where $\chi ^{(2)}$ is the second-order nonlinear susceptibility, and $\varepsilon _0$ is the permittivity of free space. However, if both interacting waves have the same circular polarizations, as in the case of using an electric field of either $E_0( {\hat e_x \pm i\hat e_y} )/\sqrt 2$, where $\pm$ means left or right circular polarization, respectively, the nonlinear polarization $P(\omega _{{\mathrm{THz}}})$ equals zero (see Supplementary Note 1).

**Fig. 1:**
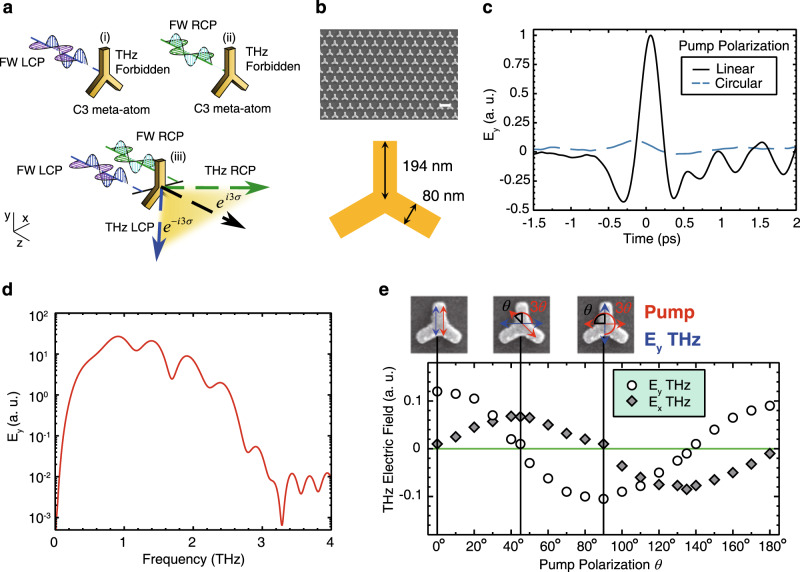
Symmetry selection rules for THz wave generation using C3 meta-atoms. **a** Symmetry selection rules for generating THz pulses from a C3 meta-atom, with both circular spin states of the pump beam required for THz generation. Excitation of the C3 meta-atom with only LCP or RCP FW's results in no generated THz signal. **b** C3 meta-atom dimensions and sample orientation. Scale bar = 550 nm. **c** Generation of a single-cycle THz pulse with linear *E*_*y*_ polarization after irradiation with near infrared femtosecond pulses with linear *E*_*x*_ polarization. **d** Corresponding frequency spectrum of the generated THz pulse, ranging from 0.6 to over 2 THz. **e** The detected *E*_*y*_ and *E*_*x*_ components of the THz field are shown relative to the rotation of the fundamental pump polarization. The maxima and minima occur due to a 3θ rotation of the THz linear polarization relative to the pump pulse as shown in the inset for the *E*_*y*_ field at $\theta _{{\mathrm{pump}}} = 0^\circ ,\,{{\Delta }}\theta _{{\mathrm{THz}} - {\mathrm{pump}}} = 0^\circ ;\,\theta _{{\mathrm{pump}}} = 45^\circ ,\,{{\Delta }}\theta _{{\mathrm{THz}} - {\mathrm{pump}}} = 135^\circ ;\,\theta _{{\mathrm{pump}}} = 90^\circ ,\,{{\Delta }}\theta _{{\mathrm{THz}} - {\mathrm{pump}}} = 270^\circ$. Corresponding *E*_*x*_ peak fields occur at pump pulse polarization angles of $\theta _{{\mathrm{pump}}} = 45^\circ$ and $135^\circ$.

Nevertheless, we find here that the restriction above does not prohibit utilizing the P-B phase to control the generated THz waves. In this case, for linearly polarized excitation, decomposing the excitation source into interacting waves with opposite circular polarizations shows that the nonlinear polarization of THz waves, $P(\omega _{{\mathrm{THz}}})$ is non-zero (See Supplementary Note 1). This finding sets new selection rules for THz generation based on quadratic optical rectification on plasmonic meta-atoms. Figure 1a provides a schematic illustration of these selection rules. Assuming the meta-atom has an in-plane orientation angle of *θ* with respect to the linear polarization of the pump wave, the nonlinear dipole moment in this case will be $P_{\!- \sigma } = \alpha _0\varepsilon _0E_\sigma E_{ - \sigma }^ \ast e^{3i\sigma \theta }$, where $\alpha _0$ is the second-order nonlinear polarizability tensor of the C3 meta-atom, and $\sigma = \pm 1$ represents the LCP and RCP states of the pump wave. The nonlinear geometric P-B phase of the emitted THz wave from such a meta-atom is therefore $- 3\sigma \theta$.

### THz emission from uniform C3 meta-atoms

To verify the THz generation selection rules and the dependence between the polarization state of the pump wave and the polarization state of the emitted THz wave, we fabricated a 1 mm × 1 mm uniform plasmonic metasurface with C3 symmetry meta-atoms (see Methods for fabrication details and Fig. 1b). The meta-atoms are designed to have a resonance at ~1500 nm (200 THz), which leads to increased extinction in this region (Supplementary Fig. 2). The metasurface is excited by the femtosecond pulses with a central wavelength of ~1500 nm and pulse width of ~50 fs. However, excitation of the metasurface can also be performed over a range of wavelengths from 1100 nm to 1600 nm. Polarization-resolved time-domain spectroscopy (TDS) based on electro-optic sampling is used to measure the generated THz (see Methods and Supplementary Fig. 1). Figure 1c shows a typical time-domain measurement of the $E_y$ polarized THz field after excitation with a $E_x$ polarized near infrared (NIR) pump wave. It can be seen that single-cycle THz pulses are generated with a full width half maximum (FWHM) pulse duration of ~0.6 ps, resulting in a THz spectrum extending from 0.6 THz to over 2.0 THz (Fig. 1d). A full spatial time and frequency map can be seen in Supplementary Fig. 3. Exciting the sample with a circularly polarized pump wave results in no appreciable THz signal (Fig. 1c), as predicted by the selection rules. In terms of THz generation efficiency, the generated THz electric field is in the region of much bulkier electro-optic crystals, with a measured peak field of approximately five times less than a 0.1 mm thick ZnTe electric optic crystal source, pumped at the same wavelength and incident laser power (see Supplementary Fig. 4).

Next, the linear polarization of the pump beam was rotated and the modulation of the $E_y$ and $E_x$ components of the THz field was measured. Figure 1e shows the amplitude of both the $E_y$ and $E_x$ components of the THz field with respect to the pump polarization angle. The maximum THz amplitudes of the $E_y$ component occur at pump polarization angles of 0°, 90°, and 180° ($E_y$ polarized THz), with minima occurring at 45° and 135° ($E_x$ polarized THz). In contrast, when measuring $E_x$ THz, maxima are observed at 45° and 135°, with minima occurring at 0°, 90°, and 180°. As expected also, the phase of both the $E_y$ and $E_x$ THz field flips when moving from one maxima to the other. As illustrated in the insets of Fig. 1e, this corresponds to an exact $3\theta$ rotation of the THz linear polarization, where $\theta$ is the relative angle between the pump polarization and the C3 meta-atom orientation. Therefore, this measurement shows that the output linear THz polarization can be continuously controlled by simply rotating the linear polarization state of the pump wave.

### Generation of THz pulses with LCP and RCP polarization states

**Fig. 2:**
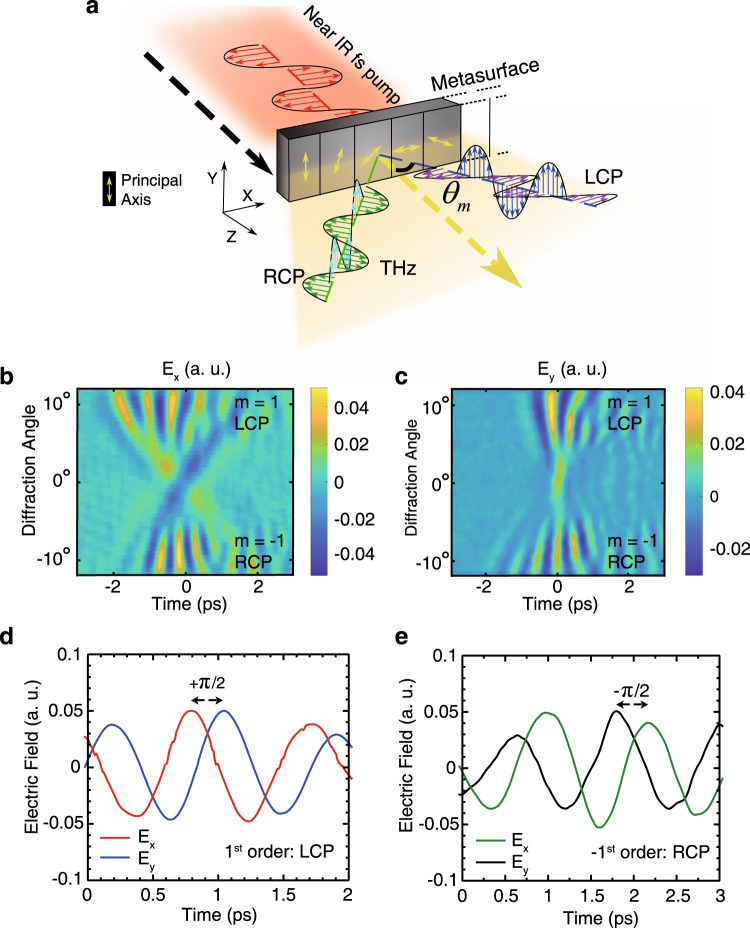
Spin-orbit interaction for THz waves. **a** Schematic illustration of a 1 mm section of the metasurface for generation of spatially separated LCP and RCP THz waves. The arrows indicate the rotation direction of the meta-atoms’ principal axis. The LCP and RCP states are diffracted to the *m* = 1 and *m* = −1 states, respectively, at angles of $\theta _m$. **b** *E*_*x*_ and **c** *E*_*y*_ components of the THz temporal fields after NIR pumping, showing multicycle pulses diffracted into orders of *m* = −1 (RCP) and 1 (LCP). In the frequency domain, this part of the waveform consists of frequency components from ~2.0 THz to 2.5 THz for both *E*_*x*_ and *E*_*y*_ fields, where lower frequencies diffract to angles beyond the numerical aperture of the collection system. **d** Relative phase shift of the $E_x$ and $E_y$ fields in the *m* = 1 and **e** *m* = −1 diffraction orders for the determination of circular polarization handedness. In the case of the *m* = 1 diffraction order, a positive phase shift is observed from *E*_*x*_ to *E*_*y*_ indicating LCP light. For the *m* = −1 diffraction order, the opposite is observed, indicating RCP light.

The new selection rules also imply that expanded non-uniform arrangements of the C3 meta-atoms can be used to construct emitters that exert specific phase patterns on the generated THz circular polarizations states. For example, it can be used to simultaneously generate THz radiation and separate the two circular spin states in the spatial domain, based on spin-orbit interaction. To demonstrate this, we fabricated a 5 mm × 1 mm metasurface where the rotation angle of the C3 meta-atoms was spatially varied according to $\theta \left( x \right) = 2\pi /3(x/{{\Lambda }})$, with a lattice period ${{\Lambda }}$ of 1 mm. Owing to the symmetry of the meta-atoms, after each period, they retain their initial alignment, leading to a continuous phase gradient with 5 full cycles. Figure 2a shows a schematic of this metasurface concept. The opposite relative gradient phase accumulated by the LCP and RCP states results in opposite diffraction directions and separation of the two spin states in space. Furthermore, owing to the periodic arrangement of the meta-atoms, different THz frequencies in the highly broadband THz pulse diffract to different angles according to the Raman-Nath condition, given by $\sin \theta _m\left( {\lambda _{{\mathrm{THz}}}} \right) = m\lambda _{{\mathrm{THz}}}/\Lambda + \sin \theta _{{\mathrm{in}}}$, where $m$ is the diffraction order, $\lambda _{{\mathrm{THz}}}$ is the wavelength of the generated THz, and $\theta _{{\mathrm{in}}}$ is the angle of incidence of the NIR pump. Altogether, the result is complete spin and frequency separation of the generated THz photons in the spatial domain, which can open the door for designing new THz spectroscopy schemes.

To examine this effect, the metasurface was illuminated with $E_x$ linearly polarized pump pulses (however, for circularly polarized THz generation the input pump polarization angle is arbitrary), and the resulting $E_x$ and $E_y$ polarized diffracted THz waves were measured, as shown in Figs. 2b, c, respectively. The diffraction angles were calculated according to the Raman-Nath condition. Multicycle THz pulses are diffracted to both $m = - 1$ and *m* = 1 diffraction orders, and the equal magnitudes of their $E_x$ and $E_y$ components indicate circular polarized THz waves. Time zero is taken as the symmetrical center of the time profile pattern. The differentiation between the LCP and RCP states can be measured by examining the phase shift between the $E_x$ and $E_y$ field components. Figure 2d shows cross-sections of the $E_y$ and $E_x$ fields measured at the same diffraction point for *m* = 1, and Fig. 2e for *m* = −1 orders. For the *m* = 1 order, a positive *π*/2 phase shift between $E_x$ and $E_y$ components is observed, indicating LCP light. In contrast, for the *m* = −1 diffraction order, a negative $\pi /2$ phase shift is observed between the $E_x$ and $E_y$ components, indicating RCP light. Furthermore, at 0 ps in the central portion of the profile in Figs. 2b, c$(\theta = 0^\circ )$, a linearly polarized $E_y$ component is measured, indicating the superposition of the THz LCP and RCP states. The spatial frequency map for such pulses is shown in Supplementary Fig. 5, with a THz region of 1.5–2 THz. Only a portion of the generated THz field is collected when the angle of incidence of the pump pulses is 0°, owing to the limited numerical aperture of the optical collection system.

**Fig. 3:**
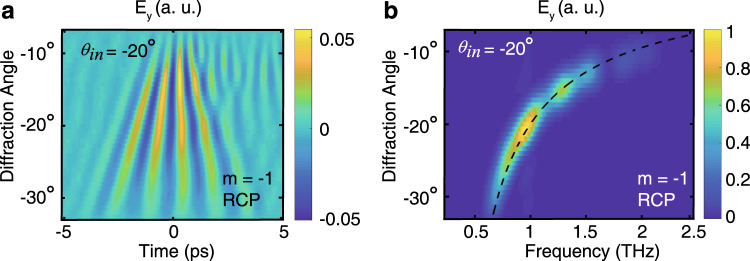
Full spatiotemporal representation of one spin-state of THz wave. **a** Measurement of the full *E*_*y*_, *m* = −1 (RCP) five-cycle diffraction order after pumping at an oblique angle of *θ*_in_ = −20°. **b** Frequency spectrum of the circularly polarized THz waves, with a spectral range from ~0.6 THz to over 2.0 THz. The dashed line shows the calculated Raman-Nath diffraction angles.

In order to collect a greater portion of the generated THz frequencies and to show the complete THz wave-packet in greater detail, the metasurface is illuminated at an oblique incident angle of −20°, which allows the entire *m* = −1 diffraction order to be directed into the collection optics^29^. A full scan of this order is shown in Fig. 3a, presenting the complete spatiotemporal structure of the five-cycle THz wave-packet, with the frequency spectrum separated in space as shown in Fig. 3b. A similar spectrum would also be observed in the *m* = 1 order. Owing to diffraction effects, higher frequency spectral components have a shorter pulse duration when compared with the lower frequency components. For example, the FWHM pulse duration is 0.22 ps, 0.35 ps, and 0.4 ps at 1.5 THz, 1 THz, and 0.8 THz, respectively, as shown fully in Supplementary Fig. 7. All together, this nonlinear metasurface allows full splitting of the spin states and frequency components of the generated THz waves.

### THz pulses with time-varying polarization states

**Fig. 4:**
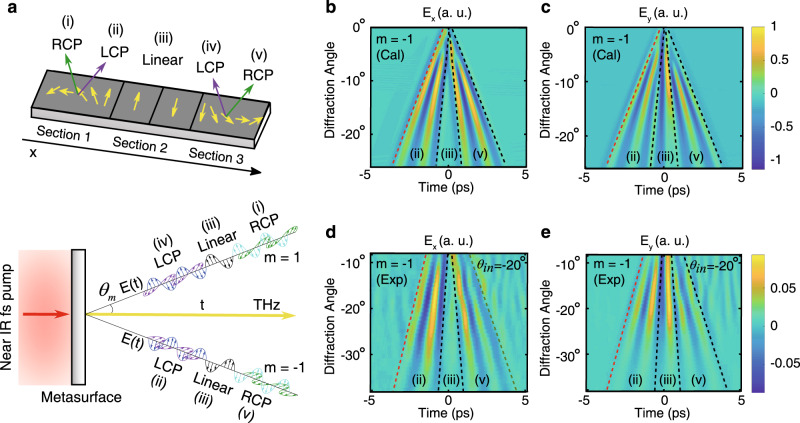
Generation of few-cycle THz waves with engineered temporal polarization dispersion. **a** Schematic of the metasurface for full THz polarization control, with regions exhibiting LCP state, RCP state, and linear polarization with opposite phase. Owing to the space-to-time mapping, two THz wave-packets are diffracted to the −1 and 1 orders, with sequential polarization profiles of (ii)/(iii)/(v) and (iv)/(iii)/(i). **b** Beam propagation simulations of the ideal temporal profile of THz waves for one complete diffraction order with *E*_*x*_ and **c** *E*_*y*_ field components from the fabricated metasurface. **d** Experimentally measured THz wave-packet with *E*_*x*_ and **e** *E*_*y*_ fields in the −1 diffraction order (the metasurface is illuminated at −20° to direct the entire diffraction order into the collection optics), showing the generation of LCP, linear, and RCP field across the generated wave-packet (see Supplementary Note 5 for LCP and RCP phase analysis). The three areas of the pulse are illustrated using dashed lines as a guide to the eye, the red dashed line indicates the region of LCP light, the black cut-out region indicates linearly polarized cycles, and the green line is the boundary of the RCP cycles. Good agreement between the simulated and experimental wave-packets is observed.

The demonstrated P-B phase concept opens the door for much more complex functionalities and control capabilities. As a proof of concept, it can be used to demonstrate complete control over the polarization state of the generated THz wave-packet, not only in space but also in time. In this way, one can generate complex few-cycle pulses with tailored temporal polarization dispersions. To demonstrate this novel concept, we designed a 5 mm × 1 mm metasurface THz emitter, where the meta-atoms are arranged into three consecutive sections (2 mm, 1 mm, and 2 mm), to generate three different polarization states in a single complex wave-packet. This concept is schematically illustrated in Fig. 4a. In section 1, the meta-atoms are gradually rotated every 100 µm with a periodic structure $\theta \left( x \right) = 2\pi /3(x/\Lambda )$ that extends for two periods, ${{\Lambda }} = 1\,{\mathrm{mm}}$, to generate two cycles of LCP and RCP THz waves diffracting in opposite directions. In section 2, the uniform meta-atoms are set in half periods with opposite vertical orientations to generate linearly polarized pulses with opposite phases. Finally, in section 3, the meta-atoms are rotated in the opposite direction to their orientation in section 1 in order to generate THz waves with opposite handedness. Owing to the direct space-to-time relation between the spatial function on the metasurface and the generated pulses in the far field, corresponding wave-packets with tailored polarization dispersion are sequentially generated in the time domain. Interestingly, owing to the symmetry of the macroscopic structure, replicas of the tailored polarization-dispersed wave-packets are diffracted in both directions (See Fig. 4a). The generated and propagating wave-packets from such a metasurface were simulated, and one diffraction order is shown in Figs. 4b, c (see Methods for simulation details). The front and rear of the wave-packet have both $E_x$ and $E_y$ components of similar strengths and opposite $E_x$ phases, whereas the middle part of the pulse has only an $E_y$ component. This observation confirms that the generated complex pulse evolves from one circular polarization (beginning with RCP in time) state to linear polarization, and then evolves further to an opposite circular polarization state (ending in LCP in time), all within five cycles. To examine this polarization behavior experimentally, the sample was excited using NIR femtosecond pulses and the emitted THz waves were characterized. The measured $E_x$ and $E_y$ components of the generated THz wave-packet are shown in Figs. 4d, e. In order to show the complete *m* = −1 diffraction order in detail, the metasurface device was illuminated with pump pulses at an oblique angle of *θ*_in_ = −20°. This is necessary to direct the entire *m* = −1 order into the collection optics. The measurements agree well with the simulations in all aspects, including the pulse angular spread in time, and the relative phase of each polarization component in the cycle, and the purely linear polarization in the middle of the few-cycle pulse, which is only seen for the $E_y$ THz field. The LCP and RCP THz fields can be confirmed by measuring the phase difference between the $E_x$ and $E_y$ components (see Supplementary Fig. 8). The frequency components of the simulated and experimental time-domain profiles are shown in Supplementary Fig. 6. Altogether the measurements confirm the theoretical prediction and show a unique generation of a complex few-cycle wave-packet that, by design, temporally alternates between different polarization states.

### Application to THz circular dichroism spectroscopy

**Fig. 5:**
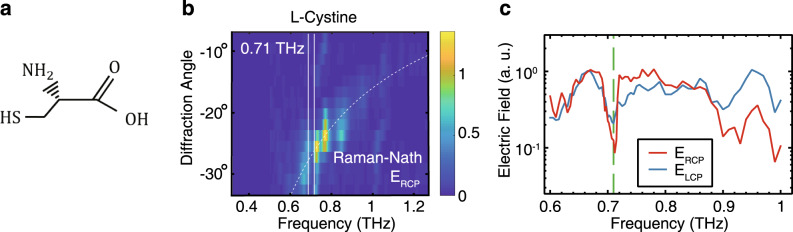
THz circular dichroism spectroscopy of l-cystine. **a** l-Cystine molecular structure. This configuration is defined as l as the amine group is to the left of the carbon chain. **b** Frequency distribution of the RCP electric field after transmission through the l-cystine powder sample, with the absorption line visible at 0.71 THz. The frequency components are separated in space owing to the Raman-Nath diffraction. The dashed line shows the calculated Raman-Nath diffraction angles. **c** Comparison of the measured frequency spectrum for both LCP and RCP THz wave-packets after transmission through the l-cystine powder sample. It can be seen that the electric field of the RCP THz wave-packet is more strongly absorbed.

The ability to generate broadband THz pulses with LCP and RCP polarization states, which can be separated in either the spatial domain (Fig. 2) or in the time domain (Fig. 4), allows the application of the metasurfaces to perform circular dichroism spectroscopy. A spectroscopic analysis of materials was undertaken using both linearly polarized pulses from the metasurface shown in Fig. 1, and from LCP/RCP circularly polarized pulses, shown in Fig. 2. The amino-acid l-cystine was chosen to perform circular dichroism spectroscopy, owing to its absorption peak at 0.71 THz. As the frequency components are instantly separated in space, the powder samples were made to a fine level of homogeneity in the spatial domain. The main absorption lines were first found using the linear THz metasurfaces from Fig. 1 (see Supplementary Fig. 9), with the primary absorption line at 0.71 THz for l-cystine. The powder sample was then illuminated with both LCP and RCP THz waves and the difference in absorption across the frequency spectrum was examined. Figure 5a shows the spatial frequency distribution of the RCP THz pulse after transmission through the l-cystine sample, with the expected 0.71 THz absorption line visible. Figure 5b shows both frequency spectrums for LCP and RCP THz pulses after transmission through the sample. For this amino acid, the RCP THz pulse is more strongly absorbed.

## Discussion

We introduce the application of P-B phase on nonlinear meta-atoms with C3 symmetry for development of unique functional THz emitters. Unlike previous manifestations of P-B phase in linear and nonlinear metasurfaces, we show that the enabling physical mechanism here is essentially different. Considering quadratic nonlinear interaction, we show that mutual interaction of the two spin states of the pump waves is essential to generate and manipulate the broadband THz waves. This understanding of the use of nonlinear P-B phase for THz generation allows unprecedented control over the polarization and phase of the THz wave-packet. We specifically show that the linear polarization state of single-cycle THz pulses can be precisely and easily manipulated just by changing the polarization state of the exciting femtosecond pulse. Furthermore, by using phase gradient nonlinear metasurfaces, both the spin states and frequencies of the generated broadband THz can be separated in the spatial domain. In addition, we show that the concept can also be used to precisely tailor the temporal polarization states of the emitted few-cycle pulse. We demonstrate this idea by introducing, for the first time, a complex few-cycle pulse that changes in the time domain from one circular polarization state to the opposite circular polarization state, through an intermediate linear polarization state. Finally, we demonstrate the application of the P-B phase nonlinear metasurface emitters for THz circular dichroism spectroscopy. All these demonstrations immediately suggest a plethora of new roles for the nonlinear P-B phase in THz science and its applications. Considering the great challenge of producing functional THz emitters, and the wealth of highly important THz applications requiring such emitters, we believe that this work can provide a highly sought way to further their implementation.

## Methods

### Metasurface fabrication

The metasurfaces used in this study were fabricated in a three-step electron beam lithography process. First, a thin positive electron resist layer (2.5% PMMA, ALLRESIST) was prepared on a cleaned ITO glass substrate using spin-coating, followed by baking at 180 °C for 3 mins. After this, the designed patterns of the metasurfaces were transferred to the prepared photoresist layer using a standard electron beam lithography process. Finally, a 30-nm-thick gold layer was deposited onto the photoresist layer using an electron beam evaporator, which finally formed the plasmonic metasurfaces after a lift-off process.

### Generation and detection of THz emission from nonlinear metasurfaces

TDS was used to measure the THz waves emitted from the fabricated metasurfaces. A femtosecond laser source (Spectra-Physics Solstice Ace) generated pulses at a wavelength of 800 nm, with a 2.0 KHz repetition rate, at 3.5 mJ per pulse, and with a 35 fs pulse duration. One percent of this output was diverted to form the probe pulse for detection, and the rest was converted by an optical parametric amplifier to form ~50 fs pump pulses with a center wavelength ~1500 nm. The pulses then went through a number of optical elements, including a half or quarter-wave plate, polarizers, and lenses, which were used to set the laser power, desired polarization state, and the size of the beam on the metasurface. After passing through the metasurface, the pump pulses were filtered out by a 5 mm thick Teflon slab. The metasurface was placed at the focal point of a parabolic mirror (*f* = 101 mm), which collected and collimated the generated THz pulses. In the collimated plane, a 7 mm slit was placed on a motorized stage, which was used to measure the beam profile. After collimation, a second parabolic mirror focused the THz waves into a ZnTe crystal. On the probe arm, the 800 nm pulses were sent to a motorized delay stage to control the temporal overlap between the probe and THz pulses. A half-wave plate and Glan polarizer were used to control both the power and the polarization of light. The probe pulse was then directed through a 3 mm hole in the second parabolic mirror to the ZnTe crystal. After passing through the ZnTe crystal, the THz electro-optic effect on the probe was measured by a set composed of a quarter-wave plate, a Wollaston prism, and a balanced photodiode. The signal from the photodiode was amplified using a lock-in detector (Standford Research Systems SR830), which was locked to the frequency of a mechanical chopper placed on the pump line and was synchronized to a sub-harmonic of the laser system. In the TDS setup used here, the amplitude of the detected pulse is dependent on the angle between the polarization state of the THz pulse and the <001> ZnTe crystal axis^39^. Maximal detection occurs when the THz pulse is perpendicular to the crystal axis, and minimum detection occurs when the pulse is parallel to the <001> axis.

### Time-domain spectroscopy of chiral molecules

In order to obtain a high-frequency resolution for the TDS analysis of the chemical samples, in the time domain the delay stage is scanned over a total corresponding time of 60 ps, which gives a frequency resolution $\Delta f = 0.02\,{\mathrm{THz}}$. Before transformation to the frequency domain, the data is processed through the application of a Gaussian apodization window. The uniform powder samples were mounted in a 3d-printed holder which was fabricated using Polyethylene, which is highly transparent in the THz region. The powdered amino-acid samples were obtained from Sigma Aldrich.

### THz propagation simulations

The simulations were based on the beam propagation technique and performed using MATLAB. The broadband pulse was defined by a spectrum matching the measured THz signal. According to the rotational orientation of the meta-atoms, the spectral amplitudes and signs of the electric field components were defined for each frequency on the metasurface plane and set to zero outside the metasurface. The spatial Fourier components of the field *E*(*k*_*x*_) were propagated along the propagation direction, *z*, for each temporal frequency, *f*, by phase addition of *k*_*z*_*z*. The collimation of the beam by a parabolic mirror was simulated by phase addition. The propagated spatiospectral structure was then used to reconstruct the spatiotemporal field by inverse Fourier transform in the time and space domains.

## Supplementary information

Supplementary Information

## Data Availability

The data that support the plots within this paper and other findings of this study are available from the corresponding authors upon reasonable request.
