# Supplementary material for: Functional THz emitters based on Pancharatnam-Berry phase nonlinear metasurfaces
Source: Nat Commun. 2021 Jan 4;12:30. doi: 10.1038/s41467-020-20283-0 (PMC7782718; doi:10.1038/s41467-020-20283-0)
Supplement: Supplementary file 1 — Supplementary Information [file 41467_2020_20283_MOESM1_ESM.pdf]

Supplementary Information for

Functional THz Emitters based on Pancharatnam-Berry Phase Nonlinear Metasurfaces

McDonnell et al.

Supplementary Note 1

Nonlinear Geometric P-B Phase and THz Radiation

Considering quadratic nonlinear interaction, for a gold plasmonic meta-atom with three-fold (C3) rotational symmetry, the second nonlinear susceptibility tensors we need to consider are *yyy* = -*yxx* = -*xxy* = -*xyx* =χ_1 (C_{3v}) or *xxx* = -*xyy* = -*yyx* = -*yxy* = χ_2 (C_{3h}), depending on the direction of the fast axis of the meta-atom. THz waves can be generated by the gold meta-atom by using second-order optical rectification. The frequency of the THz wave is written as $\omega_{THz} = \omega_1 - \omega_2$, where $\omega_1$and ω_2 are the frequencies of the two interacting waves, respectively. The polarization of the THz wave is given by $P(\omega_{\mathrm{THz}}) = \varepsilon_0 \chi^{(2)} E(\omega_1) E^*(\omega_2)$. Now, we assume a C3 meta-atom in which the x and y components are as follows:

$$P_x(\omega_{\mathrm{THz}}) = -\varepsilon_0 \chi_1 [E_y(\omega_1) E_x^*(\omega_2) + E_x(\omega_1) E_y^*(\omega_2)] \qquad \text{Eq (1)}$$

$$P_y(\omega_{\mathrm{THz}}) = -\varepsilon_0 \chi_1 [E_x(\omega_1) E_x^*(\omega_2) - E_y(\omega_1) E_y^*(\omega_2)] \qquad \text{Eq (2)}$$

If both interacting waves are entirely left or right circularly polarized (LCP and RCP), we have $E_x(\omega_1) = 1, E_y(\omega_1) = \pm i$ and $E_x(\omega_2) = 1, E_y(\omega_2) = \pm i$, and then $P_x(\omega_{\mathrm{THz}}) = P_y(\omega_{\mathrm{THz}}) = 0$. Thus the generation of THz waves is forbidden when the two waves have the same circularly polarized states.

When the incident wave is linearly polarized, from a superposition of left and right circularly polarized waves, i.e., $E_x(\omega_1) = 1, E_y(\omega_1) = i$, and $E_x(\omega_2) = 1, E_y(\omega_2) = -i$, we get $P_x(\omega_{\mathrm{THz}}) = -2i\varepsilon_0\chi_1, P_y(\omega_{\mathrm{THz}}) = -2\varepsilon_0\chi_1$. Then the nonlinear polarization of the THz wave is $P(\omega_{\mathrm{THz}} = \omega_{\mathrm{LCP}} - \omega_{\mathrm{RCP}}) = -2i\varepsilon_0\chi_1(\hat{e}_x - i\hat{e}_y)$, which means the THz wave has right circular polarization state. Similarly, if the circular polarization states of both waves are reversed, we have $E_x(\omega_1) = 1, E_y(\omega_1) = -i$, and $E_x(\omega_2) = 1, E_y(\omega_2) = i$. Then the non-linear polarization is given by $P(\omega_{\mathrm{THz}} = \omega_{\mathrm{RCP}} - \omega_{\mathrm{LCP}}) = 2i\varepsilon_0\chi_1(\hat{e}_x + i\hat{e}_y)$, which corresponds to a THz wave with an LCP state.

If the fast axis of the C3 meta-atom has an in-plane orientation angle of θ, the nonlinear dipole moment of the THz wave can be represented by the following relationship: $p = \alpha_\theta E_\sigma E^*_{-\sigma}$,where α_θ is the second-order nonlinear polarizability tensor of the meta-atom. In the

local coordinate of the C3 meta-atom, where the local coordinate axes are rotated by an angle of θ with respect to the laboratory frame, the pump and signal waves acquire a geometric phase due to the spin-rotation coupling effect: $E(\omega_1) = E_\sigma e^{i\sigma\theta}$, $E(\omega_2) = E^*_{-\sigma} e^{-i\sigma\theta}$. If the nonlinear polarizability in the meta-atom's local frame is defined as α_0, then the nonlinear dipole moment in the local frame is given by $p_{-\sigma} = \alpha_0 \varepsilon_0 E_\sigma E^*_{-\sigma} e^{2i\sigma\theta}$. After transformation back to the laboratory frame, the nonlinear dipole moment is given by

$$p_{-\sigma} = \alpha_0 \varepsilon_0 E_\sigma E^*_{-\sigma} e^{2i\sigma\theta} e^{i\sigma\theta} = \alpha_0 \varepsilon_0 E_\sigma E^*_{-\sigma} e^{3i\sigma\theta}, \quad \text{Eq (3)}$$

Where $3\sigma\theta$ represents the nonlinear geometric P-B phase of the THz wave.

Supplementary Note 2

Linear and THz Responses of the Plasmonic Metasurfaces

The experimental setup for THz measurements is described in the Methods section of the manuscript. In Supplementary Figure 1 we provide illustration of the THz generation and emission scheme. The THz spatial profiles shown in the manuscript were measured in the collimated plane by raster scanning with the moving slit which had a width of 7 mm.

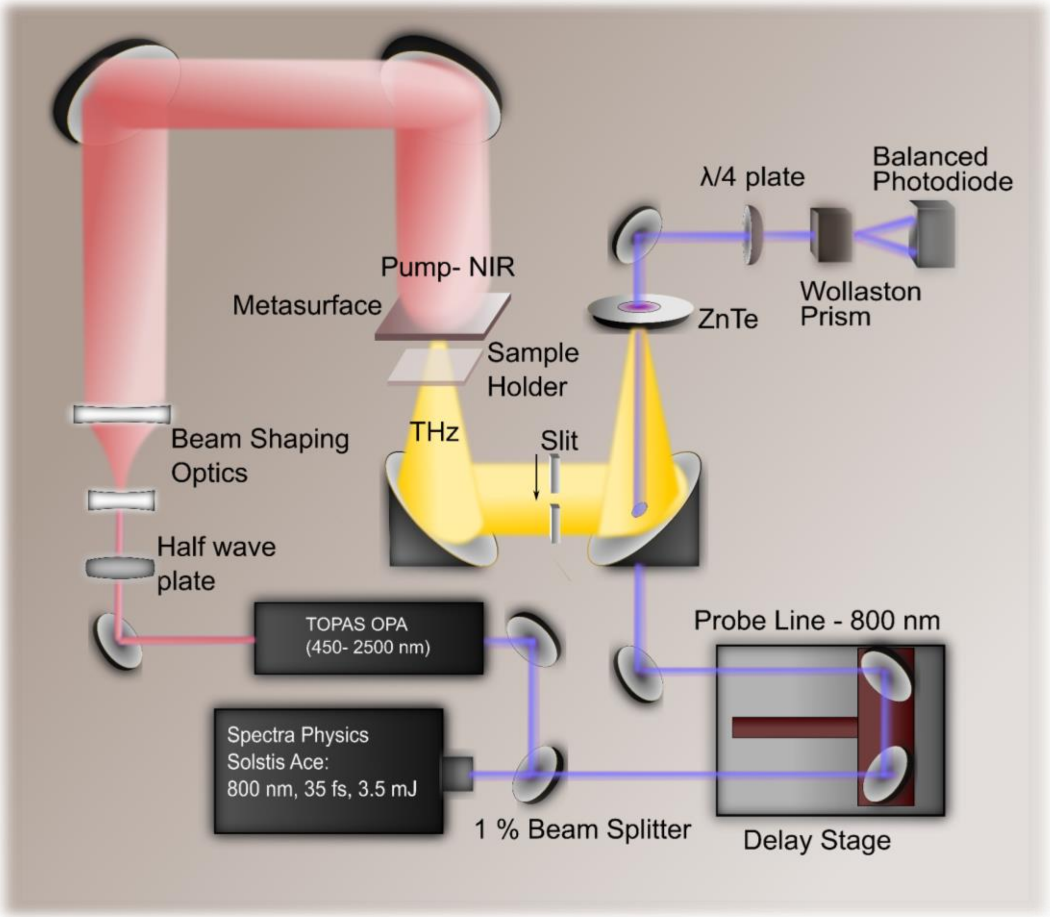

Supplementary Figure 1. **Time domain spectroscopy setup.** Schematic of the time domain spectroscopy setup used for the generation and detection of THz waves from P-B metasurfaces.

The signal to noise ratio for the generated field from the C3 metasurface can be calculated as the average electric field strength divided by the standard deviation of the peak signal[1], and is approximately 35. The dynamic range of the system is calculated to be 44, wherein the peak electric field strength is divided by the root mean squared value of the noise floor. In this case the signal is not optimized towards SNR. Significant improvement in the SNR could be achieved through increasing the time resolution of the scan, however it is not necessary for the scope of this study.

The measured linear response and the associated THz electric field response of the C3 metasurfaces are shown in Supplementary Figure 2. A broad extinction region can be noted from 1100 nm to 1600 nm, which translates into a broad THz electric field response from the

metasurface, with the measured excitation wavelengths of 1300 nm-1600 nm giving an appreciable THz response.

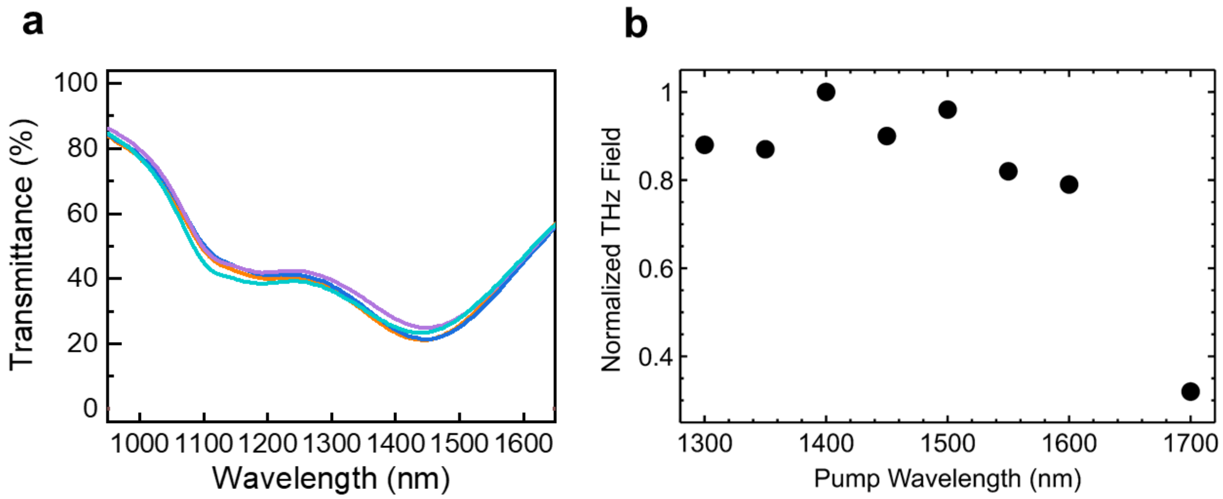

Supplementary Figure 2**. Linear response of C3 meta atoms in the near-IR range. a,** Transmittance through four C3 metasurfaces from 950 nm to 1650 nm, showing a broad absorption range from 1100 nm-1600 nm. **b,** the corresponding THz emission in overlapping region of the laser source used in this study region.

In Supplementary Figure 3 the space resolved time and frequency profiles of the generated $\hat{y}$ THz from a uniform C3 metasurface in the collimated space is shown. The beam diameter at collimation is approximately 20 mm. The dashed line in Supplementary Figure 3b show the calculated frequency dependent Raman-Nath diffraction profile from a 5 mm wide metasurface.

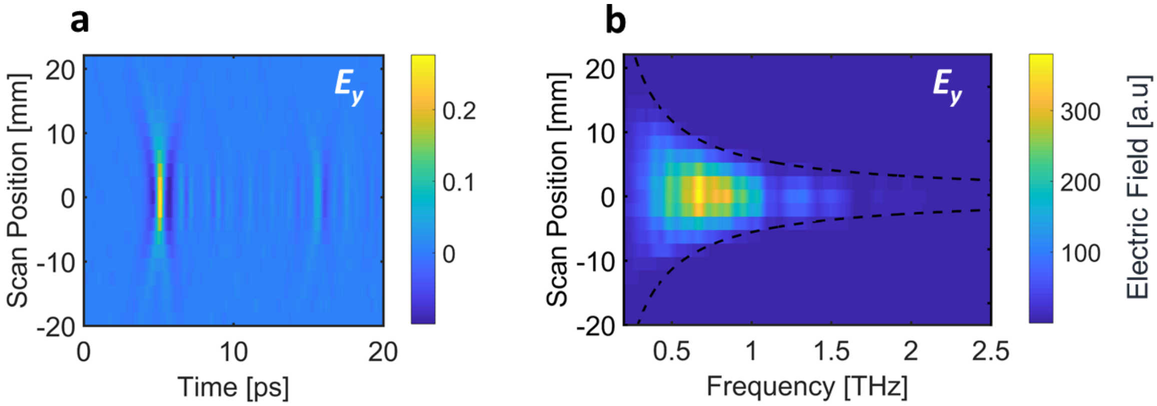

Supplementary Figure 3. **Space resolved time and frequency domain profiles for THz generation from a C3 metasurface. a,** Space resolved time domain profile of the generated THz profile, with a FWHM pulse duration of approximately 0.6 ps. **b,** Space resolved frequency profile of THz pulse. The dashed black lines show the frequency dependent Raman-Nath diffraction profile for a 5 mm wide metasurface.

The efficiency of the C3 uniform metasurface (shown in Fig. 1b) was examined by comparing the generated THz electric field to a 0.1mm thick ZnTe electro optic crystal, after pumping both sources with 1500 nm femtosecond pulses at a laser power of 25 mW. Supplementary Figure 4 shows the two electric field traces, with peaks of 0.06 and 0.28 for the C3 and ZnTe metasurface, respectively, which is an approximate 5 times relative efficiency. It must be noted that at 1500 nm the pump and the THz waves in the ZnTe are not optimally phase matched, which may cause a reduction the generated THz signal. However, since the crystal that we used is 0.1mm thick and the coherence length in this case is larger than 0.2 mm the reduction in efficiency is not significant in this case.

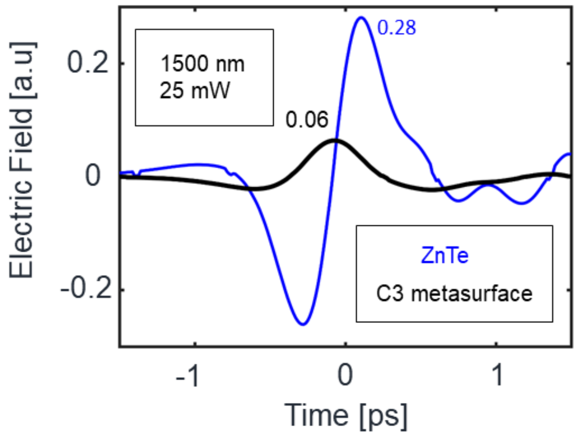

Supplementary Figure 4. **Comparison of the THz electric field strengths from the C3 metasurface and a ZnTe electro optic crystal under similar irradiation conditions.** Electric field time traces after pumping a C3 uniform metasurface and a ZnTe electro-optic crystal at a wavelength of 1500 nm and a laser power of 25 mW. The peak fields are 0.06 and 0.28, respectively.

Supplementary Note 3

Spatial Frequency Maps for the Circular and Composite THz Wavepackets

The spatial frequency map for the time domain electric fields of the LCP and RCP THz pulses (Fig. 2c) is shown in Supplementary Figure 5. The frequency components of each portion extends from 1.5 THz to 2.5 THz. The complete field is not collected due to the limited numerical aperture of the collection optics.

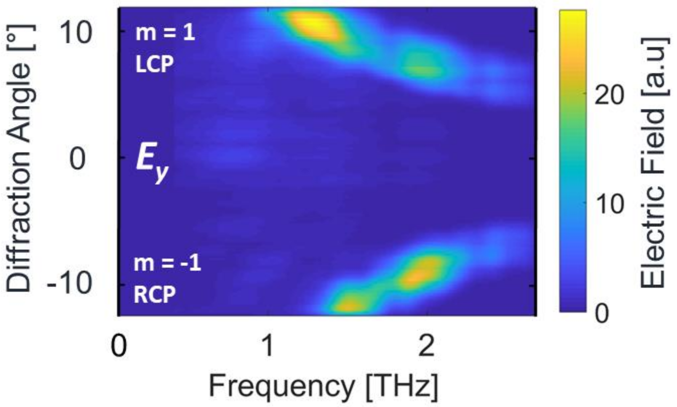

Supplementary Figure 5. **THz diffraction for pump pulses at 0º.** Spatial frequency map of the E_y LCP and RCP electric field components for the time domain electric field shown in Fig. 2.

Supplementary Figure S6 also shows the spatial frequency domain maps for the time domain profiles of complex polarization profiles shown in Figs. 4c and 4e. The experimental frequency component is narrower than the simulation, this is due to the limited numerical aperture (NA) of the experimental optics system, which results in the cut-off of the higher frequencies above 1.5 THz.

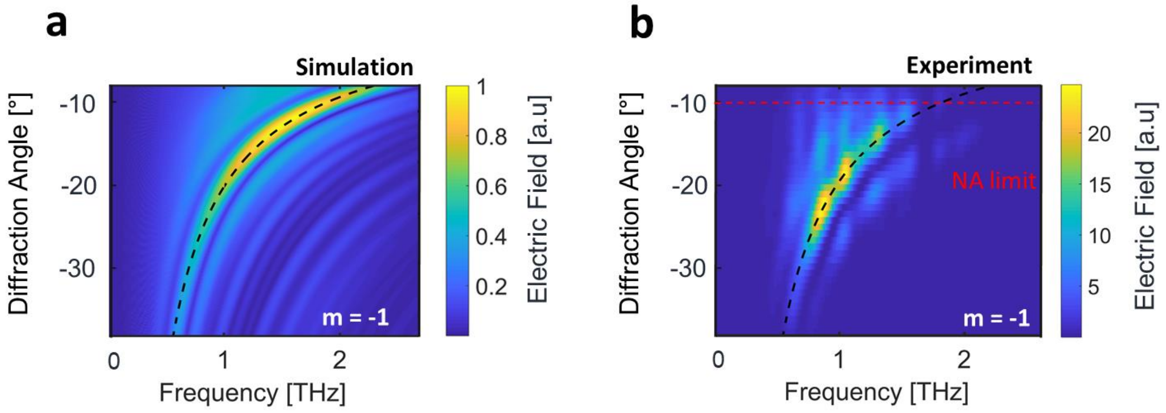

Supplementary Figure 6. **Frequency Map of the simulated and experimental complex polarization Profile. a,** Simulation of the frequency profile from the metasurface designed in Fig. 4. **b,** Experimental frequency profile taken from the Fourier transform of the time domain profile shown in Fig. 4e. The dashed black line indicates the calculated Raman-Nath diffraction angles for each frequency component. The dashed red line is used to illustrate the spatial overlap points

between the simulation and experimental data. Frequencies above 1.5 THz are cut due to the limited NA of the optical collection system.

Supplementary Note 4

Effect of Diffraction on the Pulse Duration of THz Frequency Components

The effect of diffraction on the frequency components of the RCP pulse was examined from Fig. 3. In Supplementary Figure 7 three time slices across the pulse profile are shown, which correspond to frequencies of 1.5 THz, 1.0 THz and 0.8 THz. Lower frequencies which experience a larger diffraction angle have a broadened pulse duration compared to higher frequencies.

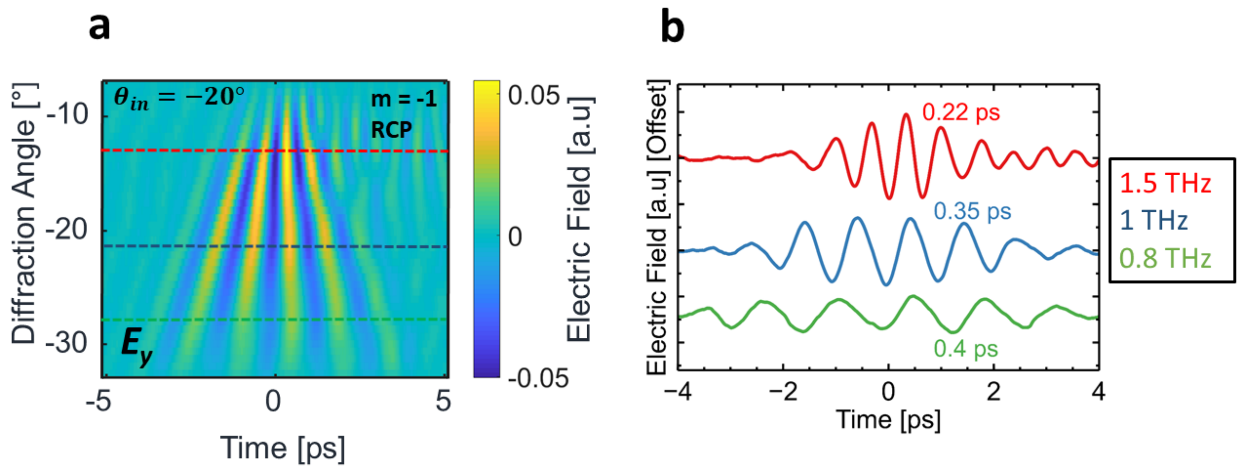

Supplementary Figure 7. **Effect of Diffraction on the Pulse Duration of the frequency components. a,** Time Domain profile of the RCP THz pulse with **b** the corresponding time slices for three frequency components at 1.5 THz, 1.0 THz and 0.8 THz. Broadening of the THz pulse with diffraction angle is observed, with lower frequencies undergoing more broadening.

Supplementary Note 5

Determination of the Handedness of Circularly Polarized THz Composite Pulse

In Fig. 4 of the manuscript, the fabricated metasurface was designed to emit a THz wavepacket consisting of a double cycle RCP polarized region, two single cycle linearly polarized pulses with opposite phase, and finally a double cycle LCP region. In order to confirm the

theoretical design and the simulation of the metasurface, the LCP and RCP regions can be confirmed by examining the phase difference between the E_x and E_y field components in a horizontal time slice across the beam profile, as shown in Supplementary Figure 8.

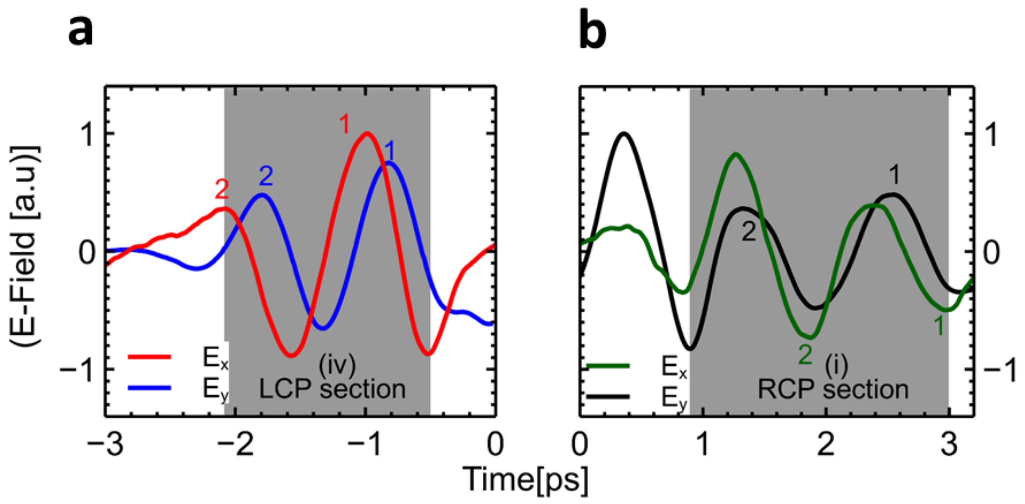

Supplementary Figure 8. **Relative phase shift of circularly polarized field components for the metasurface with engineered temporal polarization dispersion (Fig. 4 in the main text). a,** Horizontal time slice of the (iv) section of the beam profile in Fig. 4. As expected for LCP light the E_y field leads the E_x in the region of $\pi/2$. **b,** Corresponding time slice in the (i) region, with the E_x field leading E_y, as expected from RCP light.

Supplementary Note 6

Time Domain Spectroscopy using Linearly Polarized THz Pulses

The spectra of Lactose and L-Cystine were also extracted using the metasurfaces shown in Fig. 1, which generate linearly polarized THz pulses. As expected the typical strong absorption peaks is noted at 0.71 THz for L-Cystine. A reference sample of Lactose is also shown, with peaks at 0.53 THz and 1.38 THz.

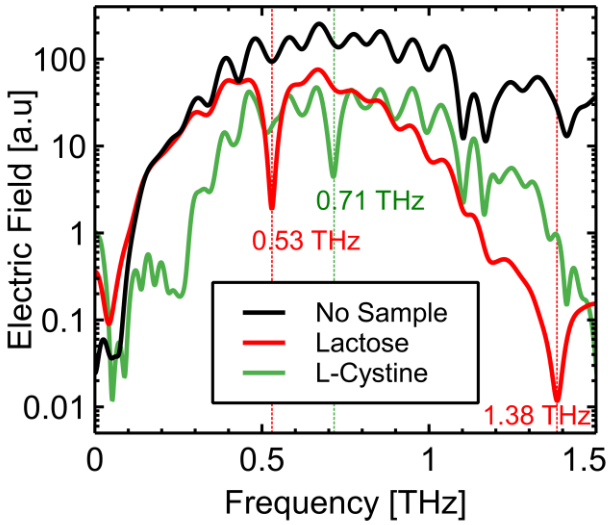

Supplementary Figure 9. **Abosprtion sprectra of Lactose and L-Cystine for linealy polarized THz pulses.** Spectra after transmission through Lactose and L-Cystine, with absorption peaks at 0.53 and 1.38 THz for Lactose, and at 0.71 THz for L-Cystine.

Supplementary References

1. Naftaly, M. & Dudley, R. Methodologies for determining the dynamic ranges and signal-to-noise ratios of terahertz time-domain spectrometers. *Opt. Lett.* **34,** 1213–1215 (2009).
